# Using Participatory Learning & Action research to access and engage with ‘hard to reach’ migrants in primary healthcare research

**DOI:** 10.1186/s12913-015-1247-8

**Published:** 2016-01-20

**Authors:** Mary O’Reilly-de Brún, Tomas de Brún, Ekaterina Okonkwo, Jean-Samuel Bonsenge-Bokanga, Maria Manuela De Almeida Silva, Florence Ogbebor, Aga Mierzejewska, Lovina Nnadi, Evelyn van Weel-Baumgarten, Chris van Weel, Maria van den Muijsenbergh, Anne MacFarlane

**Affiliations:** 1 Discipline of General Practice, School of Medicine, National University of Ireland, Galway, Ireland; 2 Department of Primary and Community Care, Radboud University Medical Centre, Nijmegen, The Netherlands; 3 Pharos, Centre of Expertise on Health Disparities, Utrecht, The Netherlands; 4 Australian Primary Health Care Research Institute, Australian National University, Canberra, Australia; 5 Graduate Entry Medical School, University of Limerick, Limerick, Ireland

**Keywords:** Migrants, User-Involvement, Meaningful engagement, Participatory research, Primary healthcare, Peer researchers, Guidelines

## Abstract

**Background:**

Communication problems occur in general practice consultations when migrants and general practitioners do not share a common language and culture. Migrants’ perspectives have rarely been included in the development of guidelines designed to ameliorate this. Considered ‘hard-to-reach’ on the basis of inaccessibility, language discordance and cultural difference, migrants have been consistently excluded from participation in primary healthcare research. The purpose of this qualitative study was to address this gap.

**Methods:**

The study was conducted in the Republic of Ireland, 2009 – 2011. We developed a multi-lingual community-university research team that included seven established migrants from local communities. They completed training in Participatory Learning & Action (PLA) - a qualitative research methodology. Then, as trained service-user peer researchers (SUPERs) they used their access routes, language skills, cultural knowledge and innovative PLA techniques to recruit and engage in research with fifty-one *hard-to-reach* migrant service-users (MSUs).

**Results & discussion:**

In terms of *access*, university researchers successfully accessed SUPERs, who, in turn, successfully accessed, recruited and retained MSUs in the study. In terms of *meaningful engagement*, SUPERs facilitated a complex PLA research process in a language-concordant manner, enabling inclusion and active participation by MSUs. This ensured that MSUs’ perspectives were included in the development of a guideline for improving communication between healthcare providers and MSUs in Ireland. SUPERs evaluated their experiences of capacity-building, training, research fieldwork and dissemination as positively meaningful for them. MSUs evaluated their experiences of engagement in PLA fieldwork and research as positively meaningful for them.

**Conclusions:**

Given the need to build primary healthcare ‘from the ground up’, the perspectives of diverse groups, especially the hard-to-reach, must become a normative part of primary healthcare research. PLA is a powerful, practical ‘fit-for-purpose’ methodology for achieving this: enabling hard-to-reach groups to engage meaningfully and contribute with ease to academic research. PLA has significant potential to become a ‘standard’ or generic approach in building community-based primary health care. Community–university partnerships have a significant role to play in this, with capacity to radically influence the shape of healthcare research, expanding the research agenda to incorporate the views and needs of hard-to-reach and vulnerable populations.

## Background

Communication between professionals (service-providers) and patients (service-users) in primary care is essential in securing a full understanding of patients and their backgrounds. Communication skills are a core competency for general practitioners [1] and necessary to build trusting relationships and achieve optimal health outcomes [2–4]. Communication touches on social and cultural values, and specific communication problems arise in cross-cultural general practice consultations when service-users and general practitioners experience language and culture barriers [5, 6]. These problems arise particularly in the care of undocumented migrants, refugees, people seeking protection (asylum-seekers) and low-income economic migrants, and persist over time and across international settings [7]. Guidelines have been developed to address this but uptake in daily practice is low [8, 9]. This may be because migrant service-users’ perspectives are seldom included in research to inform guidelines [10, 11]. In fact, the inclusion of all key stakeholders’ perspectives [12, 13] is central to the development of relevant ‘bottom-up’ health initiatives [14–17].

Participatory research (PR) [18–20], participatory action research (PAR) [21–23] and community-based participatory research (CBPR) [24–27] are used to engage stakeholders in ‘bottom-up’ primary healthcare research [15, 18, 28–32]. However, within this, we could not locate a detailed practical description of how to develop a guideline in partnership with migrant service-users. Recent meta-scoping reviews [33, 34] and a critical interpretive synthesis [10] in the field of Public and Patient Involvement (PPI) indicate that there are many types and levels of service-user involvement in healthcare research, ranging from participants as passive subjects of a study to participants actively collaborating in research design and conduct. We were aiming for active collaboration with migrants via an accessible, inclusive research process [35]. We chose PLA because it shares the democratic ethos of the approaches mentioned above and promotes active participation [36] by those who might not readily perceive themselves as experts with valuable contributions to make to academic research [12, 13, 37]. PLA techniques are accessible to those with literacy (reading/writing) challenges and two of the co-authors had extensive experience of adapting, developing and applying PLA in research with hard-to-reach groups across diverse cultural settings.

We designed a participatory study that aimed to produce a guideline integrating service-users’ and service-providers’ perspectives about strategies to support cross-cultural communication between GPs and migrants. The guideline, which promotes the use of professional interpreters and language-concordant GPs, is available [38]; the study results are reported in full elsewhere [39].

In this paper, our focus is on *method* – why and how we used a PLA approach to involve migrants in primary healthcare research. Our objectives are to describe PLA methodology, mode of engagement and techniques used for enhancing migrants’ *access to*, and *engagement in* the research process, and to report their evaluation of *engagement*.

### Definitions and description of key terms

Our research focused on migrants known to experience communication difficulties in cross-cultural general practice consultations, e.g., the undocumented, refugees, people seeking protection and low-income economic migrants [7]. Migrants with these profiles could be considered ‘hard-to-reach’, although this term is fluid and contested [40, 41]. In this study, *access* means identifying, contacting, recruiting, involving and retaining hard-to-reach migrants in a research process [35]. By *meaningful engagement*, we mean an experience of research that is *collegial, inclusive* and *active* for participants and which enables their *perspectives to emerge clearly* in research outcomes [18].

## Methods

### Study setting and rationale

The ‘SUPERS’ (Service User PEer ResearcherS) study was conducted in the Republic of Ireland (ROI) from 2009 to 2011. The study was based on a partnership between the Health Services Executive’s (HSE) Social Inclusion Unit, the Discipline of General Practice, National University of Ireland, Galway (NUI G) and the Centre for Participatory Strategies (CPS), Galway. CPS is an independent research organisation specialising in PLA research training. The HSE is the national public body responsible for the provision of healthcare to those domiciled in the State, including the increased migrant population of recent years. Between 2002 and 2011, an increase of 143 % in inward migration was recorded. ROI census figures for 2011 showed that 12.6 % of the total population were non-Irish nationals, with 19.4 % domiciled in Galway city, making it the most multicultural city in the ROI [42].

The rationale for the study developed in response to an unsolved issue in the National Intercultural Health Strategy [43]. In cross-cultural medical consultations, when general practitioners (GPs) and migrants who do not share language or culture experience communication problems, what constitutes best practice? The HSE recommended a participatory research approach to involve key stakeholders in developing a guideline to identify what communication strategies work best, for whom and in what circumstances. The intention was to identify practical solutions to everyday cross-cultural communication problems. To capitalise on the diversity of migrant groups in Galway, the HSE chose Galway city and county for the research component involving hard-to-reach migrant service-users (MSUs). The study was funded by the Health Research Board and HSE Social Inclusion Unit. Ethical approval was obtained from the Irish College of General Practitioners.

### Study design: taking a PLA approach

We chose a PLA approach and methodology for this study. Rooted in interpretive and emancipatory paradigms, PLA is a form of action research. Based on the work of Robert Chambers [13, 37, 44], PLA is a practical approach to research with diverse groups where asymmetries of power may exist [18, 45]. Influenced by critical theory and international theories of development, PLA is strongly linked to social justice movements [46–48] and, as noted above, shares the core principles of participatory action research (PAR) [47] and community-based participatory research (CBPR) [49–52].

A PLA research process brings diverse stakeholders together to engage in a process of shared, enhanced learning. A PLA ‘mode of engagement’ promotes reciprocity, mutual respect, co-operation and dialogue in research encounters within and across diverse stakeholder groups [53]. PLA techniques are inclusive, user-friendly and democratic, generating and combining visual, verbal and tangible data [12, 18, 37, 54–56]: charting, ranking, mapping and assessment techniques are combined with interviews and focus groups. This encourages literate and non-literate stakeholders alike to participate in research [12]. They are seen as ‘local experts’ [13, 44] who are uniquely knowledgeable about their own lives and conditions, who bring their *implicit* knowledge to the ‘stakeholder table’ where, through discussion and dialogue, it becomes *explicit* and therefore available to the ongoing collaborative research process they are engaged in [53]. Stakeholder groups or representatives engage in PLA-brokered dialogue to identify, in a democratic manner, positive solutions to shared problems, thereby achieving agreed goals [13, 44, 53].

PLA researchers act as facilitators, enablers and brokers, rather than directors or top-down decision-makers. This promotes strong relationships of trust and rapport with stakeholders [18, 35, 57]. Throughout iterative cycles of research, the optimum scenario is to work effectively together to address project aims, to co-design and fine-tune research plans and processes. The democratic *interactive* nature of PLA allows for co-analysis of findings. Reflection and reflexivity are addressed by engaging in team debriefing, reflection and evaluation sessions [37]. Evaluation criteria are co-generated and democratically agreed, and may serve as outcome measures. In essence, to adopt a PLA approach is to opt for an inclusive and active research process designed to promote and support *meaningful engagement* by, with and for all stakeholders, especially the least powerful.

### Study design: challenges related to access and engagement

Several key factors may constitute insurmountable barriers to access and meaningful engagement with hard-to-reach migrants. When designing this specific PLA study, we had to take account of the following:

- The university researchers had no familiarity with the languages or cultures of the intended migrant research participants and could not, therefore, engage directly in fieldwork with them.
- Migrant service-users (particularly the undocumented) may be reluctant to participate in research that brings them into direct contact with the ‘establishment’, therefore standard recruitment strategies were unlikely to generate a participant group.
- Migrants may feel uncomfortable or threatened by research that is extractive in nature and evocative of an exercise of ‘power over’ them [58]; our mode of engagement needed to reflect a very different power-sharing approach from the outset.
- Among research participants, literacy abilities may range from high to low; low literacy must be addressed sensitively.
- Appropriate evaluation tools would be required to assess migrants’ experiences of engagement, and establish to what extent these were meaningful.

### Sampling and recruitment

We used purposeful and network sampling [59, 60] as is common in qualitative research; criteria used to develop sampling frame parameters are shown in Table 1. Although representativeness is not a claim qualitative studies make, our study design involved consideration of the wide range of migrants of interest domiciled in Galway city and county. As the university researchers had no familiarity with the languages and cultures of intended migrant participants, they used their community-university networks to expand the research team. They identified seven *established migrants* from various countries of origin, who spoke a range of languages, were also proficient in English and familiar with the host culture. Already trained as community interpreters, they were interested in training as PLA peer researchers (Table 1, left-hand column). They chose the acronym ‘SUPERs’ - Service User PEer Researchers.

**Table 1:** Sampling frame parameters: criteria for SUPERs and (MSUs)

SUPERs	Hard-to-reach MSUs
Established migrant, well embedded in own community, and comfortable to self-select/identify as a representative of that community	Currently a migrant, documented, seeking protection, low income, asylum-seeker, refugee or undocumented
Domiciled in Galway city or county	Domiciled in Galway city or county
Have active social and professional networks in own community, from which migrant research participants who fit recruitment parameters may be recruited (purposeful, network sample)	Have direct social or professional contact with an established migrant from the research team; alternatively, have contact via broader migrant networks with an MSU already recruited into the study by an established migrant (purposeful, network sample)
Currently proficient in English language, but with previous or continuing (personal or professional) experience of language and culture challenges in cross-cultural primary care consultations in ROI (host country)	Current or previous experience of language and culture challenges in cross-cultural primary care consultations in ROI (host country)
Interested in availing of free training in participatory research techniques; prepared to commit time and energy to training as a peer researcher to progress sampling and fieldwork with other migrants in ROI	Willing to engage in a language-concordant participatory research study to share experiences and perspectives on language and culture challenges in cross-cultural primary care consultations in ROI

The SUPERs acted as ‘safe conduits’ for the recruitment of fifty-one MSUs into the study (Table 1, right-hand column). To do this, they:

- Co-generated a sampling frame
- Co-designed and translated recruitment leaflets into their own languages
- Disseminated leaflets throughout their community-based networks
- Engaged directly with MSUs’ questions and concerns about the study.

The languages spoken by SUPERs were a key determinant of sample selection: SUPERs’ and MSUs’ languages had to match for the planned data-generation encounters. Five SUPERs recruited one MSU group each; the two Nigerian SUPERs co-recruited the sixth group. MSUs came from a variety of countries of origin and coalesced into the following six language groups: Russian, Polish, Urdu, French-Lingalan, Portuguese/Brazilian Portuguese and Edo/Igbo/Hausa/Yoruba (Nigerian participants).

### Overview of research - Phases I, II and III

Below, we outline three distinct phases of research engagement in the study [39]:

Phase I (8 months): we focussed on capacity-building for SUPERs *(4 sessions, 12 h face-to-face)*. Using PLA techniques and focus groups discussions, we built team trust and rapport; we explored relevant international literature and policy to expand SUPERs’ knowledge-base about cross-cultural communication and we mapped strategies commonly used to address challenges in cross-cultural communication (Table 2, left-hand column).

Phase II (10 months): SUPERs completed an intensive PLA training programme (*6 sessions, 28 h face-to-face, over a 3-month period*). The time and effort devoted to capacity-building and training (total 40 h, Table 2, left-hand column; Table 5) was intended to enable SUPERs to become skilled language-concordant PLA facilitators, i.e., peer researchers capable of doing PLA research in the non-dominant languages of MSUs. At the close of Phase II, during an intensive research day (*1 session, seven hours face-to-face*) SUPERs facilitated identical sequences of 7 interlinked PLA techniques (Table 2) with MSU groups to elicit perspectives on potential guideline content.

**Table 2:** Phase I, Phase II: Methods for capacity-building, training and data generation

Note: PLA techniques described below combine visual, tangible materials (pictures, photographs, phrases, Post-It notes, symbols, voting tokens, etc) with verbal interactions, such as interviews, focus group discussions, and ‘on-the-spot’ co-analysis discussions.	
**PLA techniques used** ***with*** **SUPERs during** **capacity-building** *(4 sessions, 12 h)*	**PLA techniques use (mirrored)** ***by*** **SUPERs during fieldwork** ***with*** **MSUs** *(Intensive research day: 1 session, 7 h)*
**Ice Breaker** Interactive group activity *Rationale*: - To reduce interpersonal barriers - To build trust and rapport in research team at outset of capacity-building processes **Co-generated Ground Rules** Democratic decision-making group activity *Rationale*: - To encourage active participation by SUPERs in PLA research activity - To promote inclusion and encourage co-ownership of PLA processes - To balance power dynamics between university researchers and SUPERs - To promote empowerment of SUPERs **Timelines (individual)** Visual map and verbal narrative of university researchers’ and SUPERs’ personal and/or professional ‘journeys’ that led to participation in the study *Rationale*: - To develop deeper trust and rapport - To bond the community-university research team - To promote inclusion - To balance power dynamics **PLA-style focus group discussions** Focus group discussions using PLA ‘mode of engagement’ *Rationale*: - To develop a shared knowledge-base about international literature and policy regarding cross-cultural communication - To enhance team knowledge by mapping SUPERs’ knowledge about the range of communication strategies currently in use in cross-cultural consultations where language and culture barriers exist.	**Ice Breaker** Interactive group activity *Rationale*: - To reduce interpersonal barriers - To build trust and rapport between SUPERs and MSUs at outset of research process **Co-generated Ground Rules** Democratic decision-making group activity *Rationale*: - To encourage active participation by MSUs in PLA research activity - To promote inclusion and encourage co-ownership of PLA process - To balance power dynamics between SUPERs and MSUs - To promote empowerment of MSUs **PLA-style focus group discussions** Focus group discussions using PLA ‘mode of engagement’ *Rationale*: - To surface MSUs’ common and differential knowledge and expertise - To exchange and enhance knowledge within each MSU group during the research process - To assist data-generation in the form of a range of charts and maps developed by MSUs (as below: Flexible Brainstorming, Card Sort, Mapping, Direct Ranking) - To review and co-analyse data on completed charts and maps.
**Methods used with SUPERs during PLA training** *(6 sessions, 28 h)* Active, experiential ‘learning-by-doing’ training programme to equip SUPERs to facilitate a sequence of **7 interlinked PLA techniques**: **1. Ice-breakers** **2. Co-generated ground rules** **3. Flexible Brainstorming** **4. Card Sort** **5. Direct Ranking** **6. Mapping (visioning)** **7. PLA-style focus groups** *Rationale – to equip SUPERs to*: - Facilitate data generation with MSUs in a collegial inclusive manner likely to be meaningful for them - Use visual-verbal-tangible techniques to include all MSUs, especially those who might have literacy challenges - Promote co-analysis and co-ownership of research data by MSUs - Highlight MSUs valuable contribution to academic research **Co-design of PLA research protocol** Community-university research team activity *Rationale*: - To produce a standardised protocol for the conduct of fieldwork - To promote consistency and rigour in PLA process across fieldwork groups - To support comparative analysis of research findings across groups.	**PLA techniques used (mirrored) by SUPERs during fieldwork** ***with*** **MSUs** *(Intensive research day: 1 session, 7 h).* **1. Ice-breakers** (*see above*) **2. Co-generated ground rules** (*see above*) **3. Flexible Brainstorming** Interactive knowledge exchange, knowledge generation group activity *Rationale*: - Used to map and display a range of communication strategies known to be commonly used in cross-cultural consultations where language barriers exist **4. Card Sort** Categorisation exercise *Rationale*: - To explore and analyse communication strategies in terms of those considered ‘useful’, ‘problematic’, ‘non-viable’ **5. Direct Ranking** Democratic prioritisation, ranking and decision-making technique *Rationale*: - Used to identify ‘most-to-least’ acceptable communication strategies, as agreed by MSUs **6. Mapping (visioning activity) – ‘Ideal Scenario’** Visioning activity which maps or plots data on charts *Rationale*: - To visually map additional ideal strategies to create a vision for ‘best possible communication’ between GPs and MSUs, i.e. the ‘ideal scenario’. **7. PLA-style focus groups** (*see above*).

Phase III (6 months): The university researchers completed an identical sequence of 7 interlinked PLA techniques with service-providers (policy makers, interpreters, general practitioners and service planners) to elicit their perspectives. Then, stakeholder working groups (SWG) comprising a sample of service-providers, all university researchers and all SUPERS engaged in a PLA-brokered dialogue, during which SUPERs represented MSUs’ perspectives and recommendations. To close the project, one year after the intensive research day, SUPERs brought the outcome of the dialogue back to MSUs to seek consensus on content. They assessed the data, noting convergences and divergences between their perspectives and those of service-providers.

### Methods for meaningful engagement with SUPERs and MSUs: inclusive, active, collaborative research

The integrated nature and rigour of this study was built on an important interplay between methods used for training and capacity-building *with* SUPERs, and methods subsequently used *by* SUPERs to generate data *with* MSUs. All methods were designed to be collegial, inclusive, active and collaborative and to provide opportunities for meaningful engagement. Methods used with SUPERs were also designed to provide a *model* for their ‘mirroring’ engagement with MSUs. Below, we provide some examples across the three Phases of research; Table 2 provides further details.

Phase I: SUPERs’ capacity-building included collaborative co-design of ‘ground rules’ for respectful interaction. SUPERs’ mirrored this when, at the outset of the intensive research day, they invited MSUs to co-generate ground-rules for the day’s work.

Phase II: SUPERs’ PLA training was active and experiential; they ‘learned-by-doing’ how to facilitate PLA techniques for data-generation. They co-designed a research protocol to promote consistency and rigour during fieldwork. Each SUPER then used this protocol to facilitate, with his/her MSU group, identical sequences of PLA techniques, including:

- Flexible Brainstorming (knowledge-generation, knowledge-exchange)
- Card Sorts (categorisation, assessment, analysis)
- PLA-style focus groups (knowledge-sharing, knowledge-exchange, analysis)
- Direct Ranking (prioritisation, democratic decision-making)
- Mapping (visioning activity)

During the intensive research day with MSUs, these visual–verbal–tangible PLA techniques produced a range of charts, or ‘data displays’. These were arrayed on tables and walls, [37, 60] ready for co-analysis. SUPERs, using in-depth PLA-style focus group interviews, invited MSUs to review data, discuss emergent outcomes and offer analytical comments and insights. This collegial ‘on-the-spot’ co-analysis was possible because there were no language barriers, therefore no need for interpreters or translators. The analysis allowed clear results to emerge from each of the six MSU groups: a range of communication strategies they rejected and a range of strategies they recommended for consideration in the guideline. Following the intensive research day, SUPERs translated all data displays into English for detailed discussion with the university researchers, who recorded the data on computerized charts, enabling cross-comparison with other stakeholders’ data.

Phase III: The SUPERs, having co-analysed the results that emerged from MSU groups, were in a strong position to represent MSUs’ perspectives throughout the PLA-brokered dialogue with service-providers. This continued engagement ensured that ‘migrant voices’ were not lost.

These examples of SUPERs’ and MSUs’ involvement, inclusion, and collaboration in activities across the full research cycle illustrate a power-sharing approach, and interactive rather than extractive research. This helps to balance power dynamics and signal that researchers are oriented towards empowerment of participants [58, 61]. This empowerment lies at the heart of meaningful engagement.

### Methods used to evaluate experiences of engagement

At various points during their 2-year involvement in the project, SUPERs documented their experiences of engagement in capacity-building, training, research and dissemination via a range of qualitative methods (formative and summative). At the close of the intensive research day, MSUs documented their experiences of engagement via 3 interrelated methods (participatory evaluation, qualitative comment, Likert-type rating scales). Table 3 provides further details. The legend and codes below indicate primary data-sources:

- PE: **P**articipatory **e**valuations (SUPERs, MSUs)
- QC: **Q**ualitative **c**omments (MSUs)
- RS: Likert-type **r**ating **s**cales (MSUs)
- CH: **Ch**arted processes - mapping, ranking, data displays (MSUs)
- Ph: **Ph**otographic evidence (permitted) of charts, activities, interactions (SUPERs, MSUs)
- FDB: Post- **f**ieldwork **d**e**b**riefing interviews (SUPERs)
- PTAN: **P**roject **t**eam **an**alysis of research and evaluation data (SUPERs’ interviews, debriefings)
- CP: **C**onference **p**resentation data (SUPERs)
- Tr R/D: PLA **tr**ainers’ **r**eflections/**d**ebriefing notes

**Table 3:** Methods used to evaluate experiences of engagement by SUPERS and MSUs

**SUPERs’ experiences of engagement in:** - capacity-building - PLA training - PLA research design and planning - facilitation of PLA fieldwork - project team co-analysis - dissemination	**MSUs’ experiences of engagement:** - attendance at fieldwork session - active participation during fieldwork - co-analysis during fieldwork - retention to end of research cycle
***evaluated via:***	***evaluated via:***
- SUPERs’ post-training participatory evaluations (**PE**) - PLA trainer’s post-training reflection/debriefing notes (**Tr R/D**) - SUPERs’ post-fieldwork debriefing interviews (**FDB**) - Fieldwork photographs (**Ph**) - PLA trainers’ post-fieldwork reflection/debriefing notes (**Tr R/D**) - SUPERs’ project team analysis sessions (**PTAN**) - SUPERs’ conference presentation data (**CP**)	- MSUs’ participatory evaluations (**PE**) - MSUs’ quantitative (Likert-type) rating scales (**RS**) - MSUs’ qualitative comments (**QC**) - Fieldwork photographs (**Ph**) - MSUs’ charts, maps, data-displays (**CH**) - SUPERs’ post-fieldwork debriefing interviews (**FDB**)

### Evaluation criteria and analysis

The participatory evaluations mentioned above were based on a combination of *etic* and *emic* criteria [12, 62]: etic criteria are identified in advance by researchers. For example, our working definition of meaningful engagement included four hallmarks: ‘*collegiality’*, ‘*inclusion’*, ‘*active involvement in the research process*’ and ‘*emergence of participants’ perspectives in research outcomes’.* We used these as core etic criteria for participatory evaluations. Emic criteria are additional criteria that participants themselves may suggest. They emerge from shared ‘insider’ experiences of the research encounter. They often contribute criteria the team could not have anticipated. In our participatory evaluations, therefore, we invited participants to suggest emic criteria and invited critical comment on the etic criteria presented. The final agreed set of criteria formed the evaluation parameters.

Evaluation data were analysed using principles of thematic analysis [63, 64] to identify evidence of experiences of meaningful engagement on the part of SUPERs and MSUs.

## Results

Here we report on the core themes of this paper:

- Access to established and hard-to-reach migrants.
- Meaningful engagement in PLA research by established and hard-to-reach migrants.

### Accessing established migrant service-users (SUPERs)

The access strategies used by university researchers made it possible to include seven SUPERs in the study. A profile of the SUPERs, showing gender, region of origin, languages spoken and current profession is provided in Table 4. It is based on a self-administered questionnaire SUPERs co-designed with university researchers at the outset of the project. The five female and two male SUPERs were aged between 28 and 50. All but one had third-level education. Prior to their involvement in the project, all had completed the Northern Ireland Council for Ethnic Minorities (NICEM) interpreter’s training course. They described themselves as ‘up-skilling’ towards professional interpreting and/or wishing to establish formal accreditation of their existing interpreting qualifications in the ROI. They shared a strong commitment to the professionalising of interpreting.

**Table 4:** Profile of SUPERs

SUPERs’ ID Codes^2^	Gender	Country/region of origin	Languages	Current profession/area of interest/work
#3	Female	Russia	Russian	Migrant support & advocacy worker
			English	Community interpreter
#4	Female	Nigeria	Edo/Igbo/Hausa/Yoruba	Social worker
			English	Community interpreter
#5	Female	Poland	Polish	Healthcare assistant
			English	Community interpreter
#6	Male	Pakistan	Urdu	IT technician
			English	Community interpreter
#7	Male	Democratic Republic of the Congo	French	Research Associate
			Lingala	Community interpreter
			English	
#8	Female	Portugal	Portuguese	Interpreter and translator
			English	Doctoral candidate
			Spanish	Community interpreter
			French	
#9	Female	Nigeria	Edo/Igbo/Hausa/Yoruba	IT support engineer
			English	Community interpreter

### Accessing hard-to-reach migrant service-users (MSUs)

By activating their community networks, the 7 SUPERs successfully recruited 51 MSUs into the study. All 51 participated in the intensive research day and the majority returned one year later to discuss the outcome of the PLA-brokered dialogue.

Complex socio-political factors limited our ability to gather socio-demographic information about MSUs.

For example, educational background, literacy/numeracy abilities and other variables could not be established with precision – MSUs did not wish us to record this type of information. We were unable to establish how many were undocumented compared to those with refugee status. However, SUPERs’ observations, ‘insider knowledge’ and direct contact with MSUs during recruitment and fieldwork provided important insights into the multiple communication and access barriers these MSUs experienced: the vast majority did not speak the dominant language of the host country and were unfamiliar with the host culture. SUPERs noted that literacy abilities were mixed, and in some groups, low. Many MSUs lived in Direct Provision Centres and those without refugee status were precluded from legally entering the workforce. Some were undocumented and would not risk coming to the university for fear of being identified; they opted to work in their homes with their language-concordant SUPER. Team observations and (permitted) photographic evidence allowed us to establish that there was an evenly balanced male–female distribution, and migrants ranged in age from early 20s to mid-60s.

### Meaningful engagement by SUPERs in capacity-building, PLA training and research

SUPERs described their participation in research training, capacity-building and research as *meaningful* in terms of the hallmarks noted earlier. This is detailed in Table 5 (right-hand column) and illustrated by sample quotes below.

**Table 5:** Meaningful engagement by SUPERs

**Training activities** ***by*** **SUPERs**	**Impact/effect** ***on*** **SUPERs**
Participated in capacity-building activities: - 4 sessions, *12 h face-to-face* - Timeline activity to elicit past personal experiences of language barriers confirmed value and importance of experiential knowledge in PLA research	• Developed trust and rapport within combined community-university research team (**FDB**) • Empowered SUPERs by enhancing knowledge about cross-cultural communication in academic literature (**PE**)
Engaged in intensive PLA training: - 6 sessions, *28 h face-to-face* - additional piloting and practice hours	• Generated a skilled multiethnic, multilingual team capable of facilitating PLA research (**FDB**) • Supported development of perceived confidence and competence to act as peer researchers (**FDB**) (**PE**)
**Research activities** ***by*** **SUPERs**	**Impact/effect** ***on*** **SUPERs**
Facilitated sequence of PLA techniques in language-concordant manner - 1 session, *7 h face-to-face*	• Empowered SUPERs as active key ‘instruments’ in PLA research (**FDB**) • Confirmed value of language skills and cultural knowledge as means of addressing and ameliorating language and culture barriers in research (**FDB**) • Emphasised SUPERs’ ability to create inclusive research environment (**FDB**)
Applied range of mixed visual–verbal PLA techniques Used co-designed protocol document Culture-proofed PLA materials for use in various techniques	• Empowered SUPERs as researchers – visual nature of techniques ameliorated migrants’ literacy challenges during research process (**CH**) (**FDB**) • Confirmed unique value of SUPERs’ active input into co-design of protocol and culture-proofing of PLA materials (**FDB**)
Identified and trained a ‘materials manager’ to assist with use of PLA materials, data displays, photography and group management	• Empowered SUPERs as decision-makers (**FDB**)
Facilitated on-the-spot analysis with MSUs	• Confirmed SUPERs in their ability to engage in data co-analysis processes, which built confidence for subsequent team co-analysis (**FDB**)
Evaluated PLA research process Debriefed PLA research process	• Highlighted the positive relational environment SUPERs succeeded in generating during research process with migrants (**FDB**) • Enabled SUPERs to identify key strengths, learning, potential improvements (re PLA process, re own skills and competencies) (FDB) • Allowed SUPERs to identify perceived benefits of engagement for themselves and their communities (**FDB**)
Co-analysed research results	• Confirmed SUPERs’ skills in eliciting migrant perspectives during PLA fieldwork; confirmed value of SUPERs’ continuing involvement and input into research (**FDB**) (**CH**) (**PTAN**)

SUPERs recognised their unique power as language-concordant peer researchers who could achieve access and build rapport with MSUs:

*Our task was to be a bridge between our communities and the university. Some of the migrant service-users were hard to reach – people who were ‘seeking protection’ or were ‘undocumented’, so they were very afraid to join in anything official. [But] because each SUPER shared the language and culture of his or her group, communication was straightforward and comfortable for the service-users. We could chat about the research and explain how different [from questionnaires] it was [going to be]. SUPER #5 (FDB)*

PLA training and capacity-building supported their development as skilled and confident peer researchers capable of facilitating a complex PLA process:

*I was confident after I did the exercises with [PLA trainers]. We set up the room, [and] at that time I thought it would work, I’m equipped! So actually when I went in the field… I set up the room and everything worked for me*… *the pictures and the Direct Ranking…In Direct Ranking, everybody, they have their own view, and [can decide] how much they give [in votes] so they were thinking they are part of this research. SUPER #6 (FDB)*

*All the peer researchers used the same PLA techniques, so all stakeholder groups engaged in a consistent research process. Our results showed that by using PLA, we could ‘hear’ both dominant and hidden voices. SUPER #5 (CP)*

Reflecting on their experience of the fieldwork day, SUPERs talked about empowerment and potential benefits of engagement:

*[Being a peer researcher with my community,] I feel … very powerful and … when I listened to [MSUs’] views it gave me more energy … I feel that I have done a good job for [my community]… I’m hopeful, that [what happens] will be good for the community and the problems they are facing. SUPER #6 (FDB)*

### Meaningful engagement by SUPERs in PLA-brokered dialogue and dissemination

SUPERs’ meaningful engagement continued beyond the intensive research day when they represented MSUs’ perspectives in the PLA-brokered dialogue with service-providers. This influenced service-providers’ perspectives and informed research results, sometimes in unexpected ways. A notable example was how one GP, who considered the use of child interpreters acceptable in certain circumstances, learned (via SUPERs) about the serious negative implications of this communication strategy from MSUs’ perspectives, and revised her view. The GP then stated she would not use a child interpreter again in her medical practice. Another example was service-providers’ enhanced learning about using family members/friends as informal interpreters. Analysis of questions explored in fieldwork [39] revealed that, while MSUs considered this a ‘useful’ strategy (family members are readily available; act as advocates) they did not find it ‘acceptable’ *as best practice* and emphatically challenged any assumption that the strategy was ‘ok’ because they used it. On the contrary, many migrants were forced to choose this strategy, found it burdensome, suspected that family members could not have sufficient ‘emotional distance’ and worried about breaches of confidentiality [38]. SUPERs presented the MSU view that all stakeholders should avoid this strategy wherever possible. In the final guideline, both strategies (‘use of child interpreters’ and ‘use of family members and friends as informal interpreters’) were rejected by consensus [38, 39].

One year after the intensive research day, SUPERs returned to the university to facilitate workshops with MSUs – sharing and assessing the outcome of the PLA-brokered dialogue. The final content of the guideline was then agreed via consensus or democratic majority. This consolidated SUPERs’ central roles as peer researchers throughout the project and marked the closure of their research relationship with MSUs. Most importantly, SUPERs involvement to this ‘end-point’ of research activity was further evidence of their meaningful engagement throughout the entire research cycle.

The final experience of meaningful engagement by SUPERs occurred in the public sphere when several presented research results at local, national or international conferences. The following quotes provide a flavour of SUPERs’ reflections on meaningful engagement:

*To explain why I am still involved in these research projects, I have to use the word ‘passion’. If you do not have passion for these things, it will die without bringing any results. The relationships between the academic and peer researchers are not just work relationships, we are also personally connected. This makes the group very close … when you share an interest in something, you can discuss it, improve it, bring your ideas, you know, it was all generated together. SUPER #3* [CP]

*Migrants are rarely perceived as people who can contribute to society in terms of* ***solving problems*** *– they are often seen as groups that* ***are a problem****, and this makes it difficult to persuade them that their voices matter. So we had to build people’s confidence and reassure them that their experiences of language and cultural barriers in GP consultations were truly important and necessary to the research. We explained that they represented a critical stakeholder group and we needed them on board because their voices are so often missing in research about health policies, and health policies directly affect their lives. Most important of all, we developed strong trust relationships – this meant they could tell us the truth from their perspective and we would respect it. SUPER #5* [CP]

### Meaningful engagement by MSUs in PLA research

In terms of inclusion and involvement in research (key hallmarks of meaningful engagement) we first note that 51 MSUs remained involved and actively engaged throughout the intensive sequence of PLA techniques, seven hours, face-to-face in an unfamiliar environment. They produced sets of ranked strategies, including ‘ideal scenarios’ for effective cross-cultural communication. They actively participated in on-the-spot analysis and, finally, participated in evaluations to describe their experiences. Retention, therefore, is a result worthy of note.

Evaluation results are presented in Table 6 and illustrated in the quotes below.

**Table 6:** Meaningful engagement by MSUs

Research activities *by* MSUs	Impact/effec *on* MSUs
MSUs attended and participated in intensive language-concordant, culture-congruent PLA research fieldwork with SUPERs: *1 session, 7 hours face-to-face*	• MSUs were empowered as active participants in fieldwork; affirmed as ‘local experts’ whose opinions and experiential knowledge were essential to the study (**RS**) (**QC**) (**PH**) (**FDB**) (**CH**)
MSUs engaged in, contributed to, and completed a complex sequence of mixed visual/verbal PLA techniques	• Visual/verbal PLA techniques ameliorated literacy challenges and enhanced inclusion of mixed-literacy-ability migrant groups in research processes; completion of complex charts generated satisfaction among MSU participants (**CH**) (**QC**) (**FDB**) (**PH**)
MSUs produced a set of ranked communication strategies including ‘ideal scenarios’ for effective cross-cultural communication	• Sharing and enhancing knowledge allowed MSUs’ *implicit knowledge* to became *explicit*; ‘ideal scenarios’ included new strategies not currently in use ‘on the ground’; created energy and excitement during fieldwork (**RS**) (**PH**) (**CH**) (**FDB**)
MSUs actively engaged in *on-the-spot co- analysis* of results that emerged from their charts and maps	• MSUs’ analytical insights about emerging results affirmed the centrality of their expertise to the broader research endeavour; demonstrated the value and necessity of their continued participation at this stage of the research cycle – they ‘saw’ what others might not; emphasised uniqueness of their perspectives (**CH**) (**FDB**) • On-the-spot co-analysis by MSUs and SUPERs enhanced collegiality (**FDB**)
MSUs participated in post-research evaluation	• This inclusive collegial process signalled that migrants’ experiences of engaging in the research process were important to the community–university team (**PE**) (**RS**) (**QC**)

Asked to rate how *involved* and *included* they felt, MSUs recorded very high levels, with almost ‘perfect’ scores (RS). Their qualitative comments (QC) in response to the question ‘*What was it like to be invited to participate and ‘have your say’ with regard to the research topic focused on today?*’ provided further evidence:

*It was a positive experience which may help Irish people to realise migrants’ problems. (Polish speaker)*

*It was great to share knowledge and express my opinion. (Russian speaker)*

*I felt important and I hope that my opinion will count while introducing the changes. (Polish speaker)*

*I feel honoured to participate. (Portuguese speaker)*

*I have a place; I have been taken into account. (French–Lingalan speaker, Congolese)*

No negative comments were presented under these criteria.

We expected that working with language-concordant SUPERs and using PLA techniques might enable MSUs to confidently and competently bring their perspectives to the fore in the research, another key hallmark of meaningful engagement. This proved to be the case. Asked: *‘What was it like to work with your peer researcher through your own language today?’* positive comments included the following:

*It was excellent working in my mother tongue. (French-Lingalan speaker, Congolese)*

*[The peer researcher] presented information and explained everything very well. (Russian speaker)*

*It was confidential with someone like [the peer researcher] – there was freedom [to express one’s opinions]. (French-Lingalan speaker, Congolese)*

*Secure and confident. More power. (Portuguese speaker)*

No negative comments were presented under this criterion.

### Meaningful engagement by MSUs: reflections by SUPERs

Evaluations with MSUs took place at the close of the intensive research day, and responses were brief. We sought further clarification and confirmation of evaluation results by drawing from SUPERs’ fieldwork debriefing interviews, where they observed and reflected on MSU participation in PLA. This confirmed that the hallmarks of *inclusion* and *active involvement* were present and evident:

*People [MSUs] were showing an interest in it, in the beginning, when they saw the whole thing [PLA materials/charts etc.] [They said] ‘Oh! This is very different – this is not questionnaires, you know, it’s a completely different experience.’ SUPER #8*

*I was really surprised that the people [MSUs] were so willing to take part in it, and, particularly later on [during the day] they were so engaged in everything and they were so willing to do something … to create something, and really interested in the results. SUPER #5*

*The fact that they [MSUs] can be part of the research, they can be so motivated … you don’t know exactly what’s there, but by seeing the person in action, thinking and trying to participate, it’s really something striking, you know. They were motivated, empowered, you know, to all the process, the [research] study, so they were really committed. I think the process itself and the topic as well [influenced this] because they would like to make their concern heard by the authorities and by people. SUPER #7*

SUPERs also commented on how MSUs’ perspectives emerged clearly in the research:

*Just to see the participants, you know, coming out with the new strategies … they come out with brilliant ideas* … *that was really something to be taken into account, it was a surprise! SUPER #7*

### Meaningful engagement by MSUs – having a ‘final say’

As noted above, a majority of MSU’s returned to the university one year later to discuss and assess, with their language-concordant SUPERs, the outcome of the PLA-brokered dialogue. The final content of the guideline was agreed via consensus or democratic majority. MSUs reported that they were satisfied their perspectives had, in the main, ‘made it through’ into the guideline. This important opportunity to have a ‘final say’ reinforced their confidence in the PLA process: their perspectives had not been submerged beneath those of other stakeholders, but had emerged clearly in the research outcome: a number of communication strategies that were acceptable to all stakeholders for inclusion in the guideline, and a number of strategies that were rejected.

## Discussion

### Summary of findings

In this study, our use of a PLA approach and methodology enabled migrants (SUPERs and MSUs) to engage meaningfully and contribute with ease to rigorous primary healthcare research.

Our key finding regarding *access* is that expanding the research team to include and train SUPERs in PLA made it possible to bridge the ‘access gap’ to involve MSUs in the study, generating a wider-than-expected sample. SUPERs’ diverse linguistic abilities and cultural backgrounds eliminated twin barriers of linguistic and cultural dissonance. Relationships of trust and rapport stood the test of time – the majority of MSUs returned one year post-fieldwork to finalise the draft guideline. In our view, such *safe* and *sustained access* emphasises that peer researchers are an essential ‘bridge’ capable of linking hard-to-reach populations with the academy in positive, productive community–university partnerships for primary healthcare research.

Our key finding regarding *meaningful engagement* is that a PLA methodology enabled meaningful engagement on the part of SUPERs and MSUs throughout a full and complex cycle of research activities. For SUPERs, this encompassed training and capacity-building in PLA, co-design of protocol documents, fieldwork, co-analysis and representation of MSUs’ results in the PLA-brokered dialogue with service-providers. We consider it a strength of the study that meaningful engagement by SUPERs extended (post-project) to dissemination of findings at national and international conferences.

For MSUs, meaningful engagement encompassed genuine active involvement in collegial and inclusive PLA research and co-analysis. We believe this made an important qualitative difference to the study outcome. Given that language is the primary vehicle we use to express nuanced perspectives, working with language-concordant SUPERs promoted confident interaction by MSUs. This successfully empowered MSUs to share their implicit knowledge and unique perspectives about current communication strategies, encouraged them to propose innovative new strategies and enabled them to clarify what makes for best practice, all of which informed the development of a guideline for their care.

### Methodological critique – positive aspects

A major strength of this study was its innovative application of a PLA research methodology, mode of engagement, and series of techniques - rare in the field of migrant health and in primary healthcare more broadly [14]. PLA took us beyond *tokenistic* inclusion of MSUs and enabled them to make a valuable contribution to a primary healthcare research project. Using PLA also had impact well beyond the immediate aim of producing a guideline: SUPERs described feeling empowered in personal and professional spheres of their lives; MSUs noted that their involvement throughout the project broke through the isolation that many experienced on a daily basis in the host country. PLA, well facilitated [65], can have an integrating function that prompts broader social connections and enables empowerment in other social spheres.

Another key strength of this study was the commitment and motivation of the seven SUPERs. They were:

- Temperamentally suited to PLA training and mode of engagement – this enhanced rapport between SUPERs and MSUs, making PLA fieldwork effective and productive.
- Active in co-designing protocol documentation to guide fieldwork – this ensured consistent quality and rigour across MSU groups.
- Willing to use their ‘insider knowledge’ to benefit the project and their communities – this achieved safe access and retention of MSUs.
- Willing to represent MSUs’ perspectives during the PLA-brokered dialogue and in post-project dissemination.

The study was also strengthened by:

- Availability of professional PLA trainers in the research team.
- Adequate time and resources for training and capacity-building.
- Development of a strong community-university research partnership between SUPERs and university researchers.
- Commissioning and funding organisations who committed to a multi-perspectival PLA approach to primary healthcare research.

The absence of any one of these factors could present difficulties for other researchers wishing to initiate and sustain a PLA approach in primary healthcare research.

### Methodological critique – challenging aspects

- Complex socio-political factors made MSUs wary of sharing personal information; this limited the amount of socio-demographic data we could gather.
- Striving for quality and rigour while facilitating a complex sequence of seven PLA techniques in one day was demanding. SUPERs described feelings of exhaustion at the close of the day (however, they also described feelings of excitement: witnessing the positive nature of MSU participation and personal experiences of achievement as researchers).
- Pacing PLA research is an ever-present challenge: some MSUs said they would have preferred more time to complete the PLA techniques.
- We would have preferred more time for evaluation with MSUs; the brevity of their end-of-day evaluations did not produce the kind of rich illustrative quotes found in interview studies. (However, this lack was offset by SUPERS’ evaluation and debriefing data which augmented MSU data).

Perhaps the most challenging issue is the significant investment of time and resources required for PLA research. In this paper, we have tried to indicate the scale of that work and the range of tasks involved. We believe it is warranted by the quality of the outcome and the instrumental, practical and ethical gains achieved: meaningful engagement that is compliant with ethical recommendations for working with hard-to-reach groups, increased capacity for future community-engaged research, and a multi-perspectival guideline. Investment of time and resources in capacity-building may be considered overly time-consuming by some, but this notion tends to come from the academic perspective. In contrast, community participants rarely say that *their* investment of time and resources as PLA peer researchers is an over-commitment. In contrast, they point to experiences of enhanced learning, and meaningful engagement in research oriented towards a shared healthcare goal as adequate ‘payback’ for their investment. Furthermore, PLA training is not limited to use in a single research project; once trained, peer researchers can apply PLA to any primary healthcare research topic. Ultimately, this builds the capacity of communities to engage in rigorous participatory research hand-in-hand with the academy.

Finally, we acknowledge that migrant populations are heterogeneous in nature. This poses limitations on any qualitative study on the subject. Geo-political trends and various ‘push-pull’ factors (e.g., war, economic opportunity) also mean that migrant demographics are constantly in flux. Therefore, we are careful not to claim representativeness for the study outcomes, which took place in a particular location and socio-cultural context at a particular moment in time, with a specific group of migrants. However, in common with all qualitative studies, we suggest that the outcomes are worthy of note as a ‘depth’ insight into the research topic, which may prove valuable to other migrant groups in other situations.

### Findings discussed in relation to the literature: generating relevant primary healthcare research

Much primary healthcare research is currently initiated, designed and controlled by academic institutions with little or no input from hard-to-reach populations who are the intended beneficiaries [13, 35, 48]. In the literature, there are many examples of guidelines designed to address language and cultural barriers between GPs and MSUs that were developed without migrant input [10, 11]. There are also examples of challenges, problems and risks associated with accessing and including a range of hard-to-reach groups in healthcare research [35, 66–70]. These challenges are not sufficient reason to exclude them; rather, they are an incentive to identify methodologies capable of enhancing access and promoting meaningful engagement in healthcare research [29, 65] that takes account of health, socio-economic and cultural conditions. In line with the literature [71, 72], our study shows that including ‘the migrant perspective’ *is* possible, practical and feasible and that PLA is a ‘fit-for-purpose’ methodology [73] that produced a relevant guideline for migrant care [38, 39]. We believe that using PLA to ensure meaningful engagement in the ‘bottom-up’ generation of health initiatives and interventions is warranted because of the *quality*, *breadth* and *relevance* of the research outcome [28, 74, 75].

Our study also shows that PLA, as a brokering tool, had a powerful impact in terms of balancing asymmetrical power-relations among stakeholder groups (service-users and service-providers) and this produced a guideline qualitatively different from what would have emerged without migrants’ perspectives [76]. These findings are reported in full in a separate paper [39] but, in our results section, we noted some concrete examples of ‘aha’ moments whereby stakeholders altered their perspectives as a result of learning from others. Such major shifts in perspective are not readily made, but this is where PLA comes into its own – managing divergent experiences and potentially divisive views [12, 72]. In the transparent, democratic, dialogic PLA environment, stakeholders may gain an entirely new perspective which prompts them to shift position from long-held patterns of belief or behaviour [77]. In keeping with the literature, [37, 78] PLA cleared the way in this study for stakeholders to claim ownership of the resulting democratic outcome – a guideline oriented towards optimal health outcomes. The guideline also contributes to Irish healthcare policy. It clarifies, from a multi-stakeholder perspective, what constitutes ‘best-practice’ – solving the issue identified in the HSE’s National Intercultural Health Strategy. These are powerful arguments for using PLA in research designed to solve primary healthcare problems. However, further research is required to establish whether or not the inclusion of the migrant perspective in a guideline designed for migrants’ care actually strengthens it in practice.

### Developing community-university partnerships to support primary healthcare research

If community–university research partnerships are to develop to support primary healthcare research in the future, this study could be considered instructive as a model and also because it represents a *critical case* [60, 79]. Our use of PLA successfully overcame documented problems and issues related to including hard-to-reach groups in research [18, 35, 58]. If this can be done in the relatively challenging context and circumstances of our study, it must be possible elsewhere and in all kinds of less challenging contexts and circumstances. Our study presents a practical example of how community–university research teams, trained in the application of PLA, might go about generating research oriented towards better health outcomes for communities [2, 3, 14].

## Conclusions

Given the need to build primary healthcare ‘from the ground up’, the perspectives of diverse groups, especially the hard-to-reach, must become a normative part of primary healthcare research. PLA is a powerful, practical ‘fit-for-purpose’ methodology for achieving this: enabling hard-to-reach groups to engage meaningfully and contribute with ease to academic research. PLA has significant potential, therefore, to become a ‘standard’ or generic approach in building community-based primary health care.

Community–university research partnerships have a significant role to play in this; they have the capacity to radically influence the shape of healthcare research and expand the research agenda to incorporate the views and needs of hard-to-reach and vulnerable populations.

## Endnotes

^1^The term ‘community’ is not unproblematic – here, we understand ‘community’ as diverse, fluid, complex and heterogeneous. Crow G, Mah A: Research Report: Conceptualisations and meanings of 'community': The theory and operationalisation of a contested concept. Connected Communities, 2012. Arts and Humanities Research Council, Swindon, UK. http://community-methods.soton.ac.uk

^2^The anonymising codes assigned to the seven SUPERs began at #3 and ran to #9 and as shown are consistent with original project documentation.
